# Maternal protein restriction during lactation modulated the expression and activity of rat offspring hepatic CYP1A1, CYP1A2, CYP2B1, CYP2B2, and CYP2E1 during development

**DOI:** 10.1590/1414-431X20165238

**Published:** 2016-11-03

**Authors:** N. Meireles Da Costa, S.B.C. Visoni, I.L. Dos Santos, T.C. Barja-Fidalgo, L.F. Ribeiro-Pinto

**Affiliations:** 1 Laboratório de Toxicologia e Biologia Molecular, Departamento de Bioquímica, Instituto de Biologia Roberto Alcântara Gomes, Universidade do Estado do Rio de Janeiro, Rio de Janeiro, RJ, Brasil; 2 Programa de Carcinogênese Molecular, Instituto Nacional de Câncer José de Alencar Gomes da Silva, Rio de Janeiro, RJ, Brasil; 3 Laboratório de Farmacologia Celular e Molecular, Departamento de Biologia Celular, Instituto de Biologia Roberto Alcântara Gomes, Universidade do Estado do Rio de Janeiro, Rio de Janeiro, RJ, Brasil

**Keywords:** Maternal protein restriction, Offspring, CYP, Perinatal nutrition, Metabolic programming

## Abstract

Early nutrition plays a long-term role in the predisposition to chronic diseases and influences the metabolism of several drugs. This may happen through cytochromes P450 (CYPs) regulation, which are the main enzymes responsible for the metabolism of xenobiotics. Here, we analyzed the effects of maternal protein restriction (MPR) on the expression and activity of hepatic offspring’s CYPs during 90 days after birth, using Wistar rats as a mammal model. Hepatic CYP1A1, CYP1A2, CYP2B1, CYP2B2 and CYP2E1 mRNA and protein expression, and associated catalytic activities (ECOD, EROD, MROD, BROD, PROD and PNPH) were evaluated in 15-, 30-, 60-, and 90-day-old offspring from dams fed with either a 0% protein (MPR groups) or a standard diet (C groups) during the 10 first days of lactation. Results showed that most *CYP* genes were induced in 60- and 90-day-old MPR offspring. The inductions detected in MPR60 and MPR90 were of 5.0- and 2.0-fold (*CYP1A2*), 3.7- and 2.0-fold (*CYP2B2*) and 9.8- and 5.8– fold (*CYP2E1*), respectively, and a 3.8-fold increase of *CYP2B1* in MPR90. No major alterations were detected in CYP protein expression. The most relevant CYP catalytic activities’ alterations were observed in EROD, BROD and PNPH. Nevertheless, they did not follow the same pattern observed for mRNA expression, except for an induction of EROD in MPR90 (3.5-fold) and of PNPH in MPR60 (2.2-fold). Together, these results suggest that MPR during lactation was capable of altering the expression and activity of the hepatic CYP enzymes evaluated in the offspring along development.

## Introduction

Events that occur during fetal and early postnatal growth period are capable of determining permanent metabolic alterations throughout adult life (1). Maternal nutritional status during pre- and/or postnatal period has been associated with an increased susceptibility to chronic diseases in adulthood (2). Non-genetic factors that act early in life can organize or imprint physiological systems in a process known as ‘metabolic programming’ (3), operating by specific biological mechanisms at morphological, cellular or molecular levels (4,5). Most of the diseases caused by these non-genetic factors are associated with the impairment of hormonal homeostasis, such as cardiovascular disease, hypertension, obesity and type 2 diabetes (6,7). Nutritional status can also influence the metabolism of a wide range of drugs and other xenobiotics (8), and it may happen through the regulation of the expression of cytochrome P450 (CYPs) enzymes.

CYPs represent an enzyme family capable of catalyzing phase I reactions of biotransformation of a wide range of important therapeutic drugs, pre-carcinogens and other lipophilic xenobiotics, thus assuming special relevance to clinical pharmacology (9). Liver is the major organ of biotransformation and expresses most of the CYPs (10). Among the large diversity of existing enzymes and CYP families, members of CYP families 1, 2, and 3 are responsible for the metabolism of most drugs and xenobiotics (10). These three CYP families are subdivided into subfamilies that have their expression and activity regulated by distinct factors. For instance, CYP1A subfamily is inducible by polycyclic aromatic hydrocarbons, ß-naphtoflavone, and dioxin (11). CYP2B subfamily is generally associated with phenobarbital-type induction in rodents and in humans (12). CYP2E1 is involved in the metabolism of alcohols, aldehydes, and ketones, and plays a key role in gluconeogenesis from endogenous ketone bodies released in situations involving hormonal or metabolic changes associated with energy deprivation (13,14). CYP2E1 expression is increased by its own substrates, fasting and diabetes (13).

Protein malnutrition is an endemic condition in low income countries affecting, among others, many pregnant and lactating women. As aforementioned, maternal undernutrition during pregnancy and lactation can increase the predisposition of the offspring to chronic diseases in adult life, as well as alter drug metabolism capacity and susceptibility to toxic effects of xenobiotics, which may occur through an alteration in the expression of CYP enzymes. An increased knowledge of how nutritional status at early development may influence the metabolism of drugs in adults may contribute to a better understanding of that issue and to the development of intervention strategies. So, the aim of the present work was to investigate the effect of maternal protein restriction on the expression and activity of hepatic CYP1A1, CYP1A2, CYP2B1, CYP2B2 and CYP2E1 of the offspring during a period of 90 days after birth, using Wistar rats as a mammal model.

## Material and Methods

### Animals and diets

The procedures used throughout this study were approved by the Institutional Ethics Committee of the Universidade do Estado do Rio de Janeiro and are in accordance with the National Institutes of Health Animal Care Guidelines. A total of 144 Wistar rats were housed in controlled temperature rooms (23–25°C) with free access to water and were exposed to 12-h light and dark cycles. The protein restriction diet model was done as previously described by de Souza Caldeira-Filho and Moura (15). Virgin female rats were mated and the pregnant dams housed in individual cages. They were fed a standard diet containing 23% protein during gestation. Several pregnant female rats were kept in order to assure a litter of 6 male pups per lactating dam. The distribution of the pups among the lactating dams took place immediately after delivery. If a pregnant female gave birth to 6 or more male pups, 6 of them were kept with their progenitors; if otherwise, male pups were transferred from one litter to another in order to have 6 male pups per litter. After litter distribution, the lactating dams were fed with either a normal (23% protein) or protein-free (0% protein) diet during the 10 first days of lactation. The analysis performed in our study (CYPs mRNA and protein expression, and associated catalytic activities) were assessed in three independent pools of 6 male rats subjected to maternal protein restriction during lactation (MPR groups) or not (C groups). The male sex was chosen because they are less influenced by hormonal variations, and CYPs expression and activity have already been demonstrated to vary due to hormonal status (10). Both groups were fed *ad libitum* and diets were isocaloric. The composition of the diets is shown in Supplementary Table S1 (16,17). At the end of lactation (day 21), the litters were separated from dams and received a normal diet until 90 days of age. Animals were sacrificed by CO_2_ asphyxiation 15, 30, 60, and 90 days after birth. In all experiments, animals from the MPR group and the C group were matched by age.

### RNA extraction and RT-PCR

Hepatic tissue from 6 rats were pooled for each group in order to extract total RNA using Trizol^®^ (Invitrogen, USA), according to the manufacturer's instructions. Samples were then treated with DNase RQ1 RNase Free (Promega, USA), according to manufacturer's instructions, to avoid any contaminating DNA. The RNA was quantified by spectrophotometry and the integrity checked by electrophoresis on a formaldehyde agarose gel. RNA reverse transcription and PCR reactions were performed as previously described (18). PCR conditions were optimized to demonstrate that the amplification of *GAPDH, CYP1A1, CYP1A2, CYP2B1, CYP2B2,* and *CYP2E1* was in the linear range, in order to allow a semi-quantitative comparison of each gene expression between C- and MPR-group samples. The PCR products were run on a 6% polyacrylamide gel, stained with silver and analyzed using the LabImage software (USA). The semi-quantitative comparison of *CYP1A1, CYP1A2, CYP2B1, CYP2B2,* and *CYP2E1* expression between C- and MPR-group samples was performed normalizing the expression of these genes by *GAPDH* expression.

### Preparation of microsomes and enzyme assays

Hepatic tissue from 6 rats were pooled for each group in order to prepare liver microsomes, as previously described (19). Microsomal protein concentration was determined following Lowry et al. (20). Fifty micrograms of microsomal protein were used for each enzymatic assay. Benzyloxyresorufin- (BROD), ethoxyresorufin- (EROD), methoxyresorufin- (MROD), and pentoxyresorufin-*O*- dealkylation (PROD) were determined as described by Burke et al. (21). 7- Ethoxycoumarin-O-deethylase (ECOD) was performed as previously described (22). p-Nitrophenol hydroxylation (PNPH) activity was measured following Allis and Robinson (23).

### Western blotting

SDS-PAGE of microsomal proteins isolated from a pool of hepatic tissue from 6 rats of each group was performed on an 8% polyacrylamide resolving gel. After electrophoresis, proteins were electroblotted onto a nitrocellulose membrane (Trans-Blot transfer Medium, Bio-Rad, USA). The membrane was blocked and incubated with rabbit antibodies against rat CYP1A1/1A2 (1:1000), CYP2B1/2B2 (1:1000), generously donated by Dr. E.A. Shepard (University College London, UK), and CYP2E1 (1:1000), a generous gift from Dr. Peter Swann (University College London, UK). Detection of proteins was performed by using the alkaline phosphatase kit, according to the manufacturer's instructions (BioRad). The semi-quantification of protein bands was achieved by using LabImage software.

### Statistical analysis

Data presented in this study (for each group and age) derived from three independent pools of 6 male rats from MPR and C groups, i.e., the N of the statistical unit of analysis was 3. Statistical analyses were performed using the software GraphPad Prism (GraphPad Software, USA). Student's *t*-test was employed to compare mRNA and protein expression, and catalytic activity between groups at each developmental age. P<0.05 was considered to be statistically significant.

## Results

The animals from the MPR group presented a reduction of about 50% in body weight from 8 to 21 days of age compared with the animals of the same age from the C group. Similar results were achieved in a previous study using the same protein restriction model (16). During development (30 and 60 days of age), this difference diminished to about 20% (Supplementary Figure S1).

### Protein-free diet during lactation modulated the offspring hepatic *CYPs* mRNA expression mainly at adulthood

**Figure 1:**
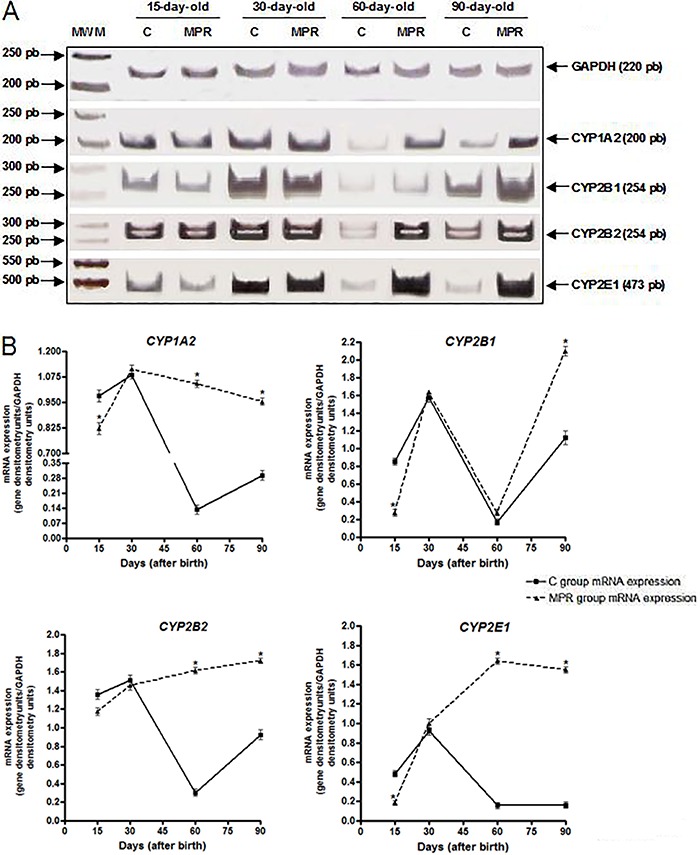
Protein-free diet during lactation modulates the offspring hepatic *CYPs* mRNA expression at adulthood. *A*, representative images of hepatic *CYP1A2*, *CYP2B1*, *CYP2B2* and *CYP2E1* mRNA expression in 15-, 30-, 60- and 90-day-old offspring from dams fed either a 0% protein (MPR groups) or a standard diet (C groups) during the first ten days of lactation, evaluated by RT-PCR in a 6% polyacrylamide gel; *CYP1A1* expression was not detected. *B*, Densitometric analysis of *CYP1A2*, *CYP2B1*, *CYP2B2* and *CYP2E1* mRNA expression. Data are reported as median and standard deviation of three biological replicates and normalized by GAPDH analysis (mRNA expression data derived from three independent pools of 6 males from each group, i.e., N=3). MWM: molecular weight marker (pEJ3 plasmid DNA digested with using Eco147II and PvuI). *P<0.05 for MPR samples compared to their C counterpart samples (Student's *t*-test).

*CYP1A1* mRNA expression was not detected in any sample even when reactions were performed over 40 cycles. Among control animals, the expression of all the other *CYP* genes evaluated was detected at all time intervals, displaying a similar profile among them, with the highest expression level being detected in 30-day-old animals (Figure 1A). Among MPR-group animals, *CYP1A2* mRNA expression was induced in 60- and 90-day-old animals (5- and 2-fold, respectively; Figure 1A and B). *CYP2B1* mRNA levels increased 3.7-fold in 90-day-old animals, whereas *CYP2B2* mRNA was increased 3.7- and 2.0-fold in 60- and 90-day-old animals, respectively. *CYP2E1* mRNA expression showed an increase in animals at all ages, with the highest induction occurring in 60-day-old rats (10-fold increase), as shown in Figure 1A and B.

### Maternal protein-free diet during lactation altered the offspring hepatic CYP2B1/2B2 protein expression levels at adulthood

**Figure 2:**
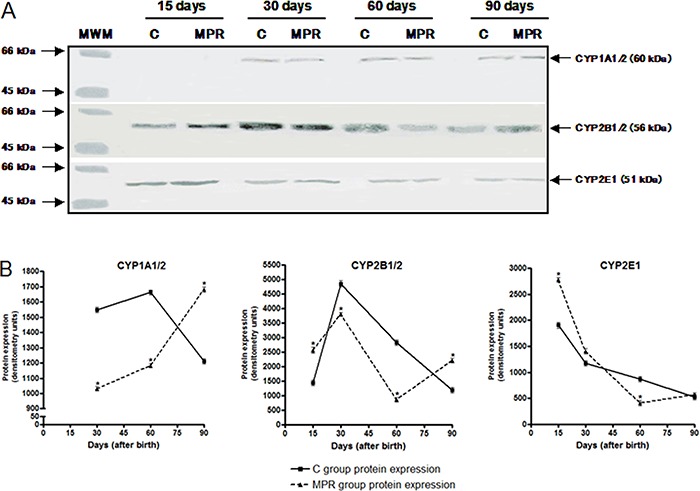
Maternal protein-free diet during lactation alters the offspring hepatic CYP2B1/2B2 protein expression levels at adulthood. *A*, representative images of hepatic CYP1A1, CYP1A2, CYP2B1, CYP2B2 and CYP2E1 protein expression in 15-, 30-, 60- and 90-day-old offspring from dams fed either a 0% protein (MPR groups) or a standard diet (C groups) during the first 10 days of lactation, evaluated by western blotting. *B*, Densitometric analysis of CYP1A1/1A2, CYP2B1/2B2 and CYP2E1 protein expression. Data are reported as median and standard deviation of three biological replicates (protein expression data derived from three independent pools of 6 male rats from each group, i.e., N=3). MWM: molecular weight marker (containing serum bovine albumin and ovalbumin). *P<0.05 for MPR samples compared to their C counterpart samples (Student's *t*-test).

Figure 2A and B represents CYP protein expression analysis by western blotting. A significant 42% decrease and 60% increase in CYP2B1/2B2 protein levels was observed in 60- and 90-day-old MPR animals, respectively. Nevertheless, no significant changes in CYP1A1/1A2 and CYP2E1 protein expression profile were found between animals from MPR and C groups at the analyzed time intervals.

### Hepatic CYP enzymatic activities profile was altered in the offspring from dams fed with a protein-free diet during lactation

**Figure 3:**
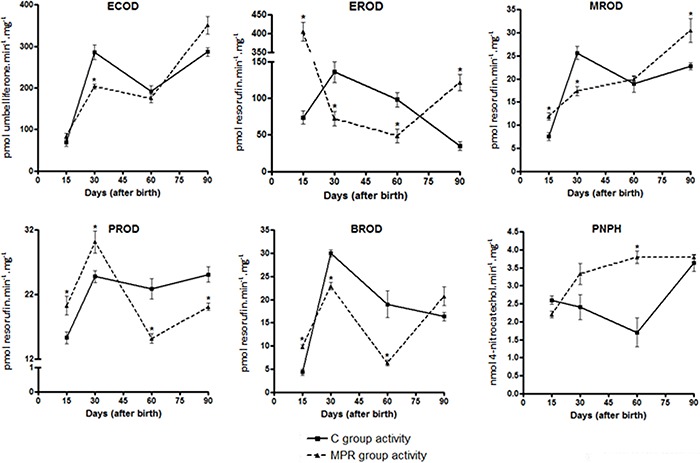
Analysis of ethoxycoumarin-O-deethylation (ECOD), ethoxyresorufin-O-deethylation (EROD), methoxyresorufin-O-demethylation (MROD), pentoxyresorufin-O-depentylation (PROD), benzyloxyresorufin-O-debenzylation (BROD) and p-nitrophenol hydroxylation (PNPH) activities measured in liver microsomes from 15-, 30-, 60- and 90- day-old offspring from dams fed either a 0% protein (MPR groups) or a standard diet (C groups) during the first ten days of lactation. Data are reported as median and standard deviation of three biological replicates (catalytic activities data derived from three independent pools of 6 male rats from each group, i.e., N=3). *P<0.05 for MPR samples compared to their C counterpart samples (Student's *t*-test).

Figure 3 and Supplementary Table 1 show a slight but significant decrease (29%) of ECOD in 30-day-old MPR animals. EROD was significantly decreased in 30- (47%) and 60- (50%) day-old, but increased in 15- (5.6-fold) and 90- (3.5-fold) day-old MPR animals. MROD was increased in 15- (1.5-fold) and 90- (1.3-fold) day-old, but slightly decreased (23%) in 30-day-old MPR rats. PROD activity was slightly decreased in 60- (33%) and 90- (20%) day-old, but increased in 15-day-old animals (1.3-fold). BROD was decreased in 30- (23%) and 60-day-old (68%) MPR animals, but increased in 15- and 90-day-old MPR animals (2.2-, and 1.3-fold, respectively). Finally, PNPH was increased (2.2–fold) in 60-day-old, and slightly decreased (17%) in 15-day-old MPR animals.

## Discussion

Early nutritional status has been considered to play a putative long term role in the predisposition to some chronic diseases (1). It has been shown by different studies using a variety of animal models that maternal nutritional condition alters the offspring's metabolism. Maternal obesity, overnutrition (e.g., diets high in fat) and undernutrition (e.g., low protein and/or calorie intake) during gestation and/or lactation result in increased insulin secretion, obesity, insulin-resistance, type 2 diabetes and cardiovascular diseases (24–26). These metabolic alterations can lead to an unbalanced exposure to non-genetic events, such as from hormones, metabolites and neurotransmitters. Especially during a sensitive period as early development, these events are capable of altering the organization of different physiological systems in a process known as ‘metabolic programming’, which can lead to the development of diseases at adulthood (1,27). In addition to the increased predisposition to the development of chronic diseases in adult life, nutritional status can also influence the metabolism of a wide range of drugs and other xenobiotics (8). CYP enzymes are capable of metabolizing a large number of important therapeutic drugs and other xenobiotics (9). However, there are no studies to date that show the effect of maternal protein restriction on distinct CYP enzymes expression and activity during different periods of the offspring life.

In this study, we showed that maternal protein restriction during early lactation in rats altered the expression and activity of hepatic CYP1A1, CYP1A2, CYP2B1, CYP2B2, and CYP2E1 of the offspring. Of note, a significant induction was observed in the expression of all *CYP* genes analyzed in this study, occurring mainly in 60- and 90-day-old rats. Nevertheless, only a discrete alteration in CYP2B1/2B2 apoprotein expression in the liver of MPR animals was detected. Similarly, the catalytic activities did not follow the exact same variations observed in the mRNAs levels of the offspring.

Noteworthy, the most adequate statistical evaluation for our study design would have been a two-way ANOVA with *post hoc* tests in order to compare the age and nutrition group parameters. Nevertheless, due to our small N (equal to 3), this test could not be performed, representing a limitation of the statistical evaluation. In addition, our major findings were in gene expression analysis. Although more advanced methodologies than the one used in this study are available to investigate gene expression, the differences detected were high enough to detect significant increases in *CYPs* expression in the offspring at adulthood. The differences observed between the mRNA expression and the CYP isoforms-associated catalytic activities may be explained in three ways. First, the effect of maternal protein restriction on hepatic CYP activities of the offspring may be caused by a post-transcriptional or post-translational regulation. The second explanation is that the performed catalytic activities are associated, but not specifically, to the analyzed isoforms. They become specific activities only in the presence of specific inducers to each CYP isoform, which considerably increase their protein expression (21). The third possible explanation for the increased mRNA expression levels of some CYP enzymes followed by a reduced associated catalytic activity lies in that it could represent a compensatory response – i.e., the tissue may be working to overcome the reduction in activity by increasing gene transcription. Nonetheless, our results are in accordance to a previous study that showed a decrease of 42, 71 and 90% in the levels of PROD, EROD and BROD activities, respectively, in 66-day-old rats submitted to a 6% protein diet during 45 days (28).

Another study evaluated the effects of maternal low protein diets on the ontogeny of CYPs and CYP reductase (CPR) activities in the rat offspring and, contrary to our results, reported a decrease in the enzymes activities at the age of 28 days and the reversion of the phenomenon at adulthood (65-day-old offspring). The experimental model was the administration of two low protein diets to rats during pregnancy and lactation and the evaluation of the effects of the nutritional change during the perinatal period on CYPs and CPR activities in male and female offspring(29). Differences between the two studies might explain the diverging results: the analyzed CYP enzymes are different making it difficult to fully compare data of the studies; in the other study, gene expression was not assessed and our major findings were in gene expression analysis; the timing and duration of the maternal protein restriction are distinct; and the type and amount of protein used also varied between the two studies. Corroborating the last explanation, it has been reported that the type and amount of protein used in maternal diets affects offspring’s microsomal protein yields (30).

Even though maternal low protein diets during pregnancy and lactation are more frequently used as experimental approaches, we took advantage of the possibility to modulate the diet composition to investigate extreme situations. The maternal 0% protein diet during the first days of lactation used in our study was based on published studies, which also reported relevant alterations in the offspring at adulthood. Of note, lower insulin secretion followed by an increase in glucose uptake was observed in the muscle of adult rats subjected to maternal undernutrition, in addition to a higher GLUT-4 translocation associated to a higher IRS-1 phosphorylation. These results suggest an increase in insulin sensitivity in adulthood, which appears to be a compensatory mechanism to the lower insulin secretion (16). The offspring from dams fed with a protein-free diet during the ten first days of lactation showed a decrease in pleural formation (50%), neutrophil migration (50%), endothelial ICAM-1 expression on pulmonary tissue, impairment in leukocyte adhesion (50%) and migration (80%) through endothelium, and altered hormones secretion with lower levels of circulating insulin (42%) and higher levels of corticosterone (34%) at adulthood, resulting in an impairment of the inflammatory response (31). Taken together, these results show that the animal model employed in our study is efficient in helping us understand the alterations produced by maternal protein restriction during lactation in metabolism, as well as in inflammatory response, of the offspring at adulthood.

Although alterations in glucose metabolism have been detected in studies using the same experimental model, diabetes and fasting cannot explain the induction observed in mRNA levels, once plasma glucose levels are normalized at 60-day-old (16). However, alterations found in gene expression and associated catalytic activities of the enzymes analyzed in the present study could be caused by metabolic or epigenetic alterations in the offspring due to maternal protein restriction during early lactation. DNA methylation is essential for imprinting mechanisms (32). Nutritional alterations in early life are capable of modifying specific cellular DNA methylation patterns, altering gene expression in specific tissues (33). Genomic DNA methylation was evaluated in the liver of rat's fetuses from dams subjected to a protein restriction diet (8%) during pregnancy and an increase in methylation was detected, due to a raise in threonine metabolism (34). Furthermore, at least three out of the five CYP enzymes analyzed in this study have been described to be epigenetic-regulated: CYP1A1 (47), CYP1A2 (35,36) and CYP2E1 (37,38). However, we did not evaluate whether the maternal protein restriction effects observed on the hepatic expression of these CYP genes in the offspring were due to alterations on the methylation pattern of their promoter regions.

In conclusion, our results showed that maternal protein restriction during lactation can alter the expression and activity of CYP enzymes of Wistar rats offspring, particularly at adulthood.

## Supplementary material

Click here to view [pdf].
